# Deciphering the Adjustment between Environment and Life History in Annuals: Lessons from a Geographically-Explicit Approach in *Arabidopsis thaliana*

**DOI:** 10.1371/journal.pone.0087836

**Published:** 2014-02-03

**Authors:** Esperanza Manzano-Piedras, Arnald Marcer, Carlos Alonso-Blanco, F. Xavier Picó

**Affiliations:** 1 Departamento de Ecología Integrativa, Estación Biológica de Doñana (EBD), Consejo Superior de Investigaciones Científicas (CSIC), Sevilla, Spain; 2 CREAF, Cerdanyola del Vallès, Spain; 3 Universitat Autònoma de Barcelona, Cerdanyola del Vallès, Spain; 4 Departamento de Genética Molecular de Plantas, Centro Nacional de Biotecnología (CNB), Consejo Superior de Investigaciones Científicas (CSIC), Madrid, Spain; The Australian National University, Australia

## Abstract

The role that different life-history traits may have in the process of adaptation caused by divergent selection can be assessed by using extensive collections of geographically-explicit populations. This is because adaptive phenotypic variation shifts gradually across space as a result of the geographic patterns of variation in environmental selective pressures. Hence, large-scale experiments are needed to identify relevant adaptive life-history traits as well as their relationships with putative selective agents. We conducted a field experiment with 279 geo-referenced accessions of the annual plant *Arabidopsis thaliana* collected across a native region of its distribution range, the Iberian Peninsula. We quantified variation in life-history traits throughout the entire life cycle. We built a geographic information system to generate an environmental data set encompassing climate, vegetation and soil data. We analysed the spatial autocorrelation patterns of environmental variables and life-history traits, as well as the relationship between environmental and phenotypic data. Almost all environmental variables were significantly spatially autocorrelated. By contrast, only two life-history traits, seed weight and flowering time, exhibited significant spatial autocorrelation. Flowering time, and to a lower extent seed weight, were the life-history traits with the highest significant correlation coefficients with environmental factors, in particular with annual mean temperature. In general, individual fitness was higher for accessions with more vigorous seed germination, higher recruitment and later flowering times. Variation in flowering time mediated by temperature appears to be the main life-history trait by which *A. thaliana* adjusts its life history to the varying Iberian environmental conditions. The use of extensive geographically-explicit data sets obtained from field experiments represents a powerful approach to unravel adaptive patterns of variation. In a context of current global warming, geographically-explicit approaches, evaluating the match between organisms and the environments where they live, may contribute to better assess and predict the consequences of global warming.

## Introduction

Annual plants exhibit contrasting life histories in different environments varying in predictability of the amount and timing of yearly precipitation. On the one hand, annuals occurring in highly unpredictable environments, e.g. deserts, are typically characterised by persistent seed banks and delayed seed germination. When environmental conditions become favourable for seed germination and seedling establishment, annuals can complete the life cycle in weeks. Hence, desert annuals reduce temporal variance in reproductive success and buffer the long-term risk of extinction in unfavourable years, representing a bet-hedging strategy in unpredictable environments [1]–[7]. On the other hand, annuals inhabiting humid temperate areas mostly behave as winter annuals because the environment is seasonal and far more predictable than in deserts. Winter annuals remain as seeds buried in the soil during summer, peak germination in autumn, overwinter as vegetative rosettes, and become reproductive in late winter or early spring [8]–[12]. Thus, bet-hedging and winter annual behaviour are regarded as adaptive life histories at the opposing margins of a gradient of environmental severity and predictability between deserts and temperate areas.

Obviously, environments are not homogeneous and annuals are expected to modify the architecture of life-history traits reflecting environmental and ecological differences among populations [13], [14]. Two traits stand out above all when annuals adjust the life history to environmental conditions: the timing of germination and flowering. Due to the short duration of the life cycle in annuals, germination timing represents the first major developmental transition influencing all posterior life-history traits [15]–[19]. In addition, flowering time is also an important trait whose variation has been shaped by natural selection to maximise reproductive success [20]–[25]. Hence, annual plants are expected to fine-tune the timing of germination and/or flowering to succeed in a given environment. Indeed, recent empirical studies showed that some annuals tend to lower germination fractions [26]–[28] or flower earlier [21], [29]–[32] as environmental stress increases, such as that caused by climate warming or increasing aridity.

The methodological approaches commonly employed to study the evolutionary consequences of variation in life-history traits of plants include common garden and/or reciprocal transplant experiments between contrasting environments. Such experiments are very powerful because they allow the assessment of the genetic differentiation among lineages from different populations for the life-history traits of concern [33]–[36]. However, an important drawback is that these experiments are very demanding and researchers generally opt for selecting an appropriate set of populations from representative environments [37]–[40]. Therefore, experiments have to sacrifice potentially relevant genetic variation, which may limit our understanding of how the adjustment between environmental factors and life-history traits works. Alternatively, correlation analysis between genetic variation in phenotypic traits and environmental variation has long represented a powerful method to detect selection along environmental gradients [41]–[45] because adaptive phenotypic variation shifts gradually across space as a result of the geographic patterns of variation in major selective pressures, e.g. climate. Given the fact that spatial patterns of variation exist in both environmental and phenotypic traits, understanding large-scale adaptive patterns of variation in life-history traits of annual plants demands geographically-explicit experimental approaches.

Here, we aim to analyse the adjustment between geographical variation in environmental factors and life-history traits of the annual plant *Arabidopsis thaliana*. We employ a collection of 279 geo-referenced populations from the Iberian Peninsula where *A. thaliana* has been studied for years due to its large genetic and environmental variation [12], [46]–[49]. Study populations represent a wide array of Iberian natural environments, encompassing humanised and wild habitats as well as low- and high-elevation locations. Each population was characterised by climate, vegetation and soil features. Genetic variation in life-history traits was evaluated by replicating one individual from each population in a common garden experiment. Experiments in natural field settings are unavoidable if we aim to obtain realistic data on genetic variation in phenotypic traits. We specifically address two main questions. First, what is the spatial autocorrelation pattern of *A. thaliana*’s life-history traits in the Iberian Peninsula? If environmental factors have shaped variation in life-history traits, we would expect to detect it by the extent of spatial autocorrelation in life-history traits given the inherent spatial variation in environmental factors. Second, what is the relationship between variation in environmental factors and life-history traits in Iberian *A. thaliana* populations? The environmental factors and the life-history traits affected by them are expected to be correlated. The results are discussed in a context of adaptive geographic variation stressing the value of extensive sampling to identify ecologically and evolutionarily important life-history traits in plants.

## Materials and Methods

### Study Organism and Source Populations

*Arabidopsis thaliana* (L.) Heyhn. (Brassicaceae) is a cosmopolitan annual plant native to Europe and Central Asia [50]. In the Iberian Peninsula, the plant is widely distributed occurring in both humanised and wild habitats [46], [48]. This self-compatible and self-fertile plant possesses a seed bank with an estimated half-life of approximately three years [12]. *Arabidopsis thaliana* can behave as a winter or spring annual [49], [51], [52] depending on the timing of germination (autumn *vs.* spring) and the state in which the plant overwinters (vegetative rosette *vs.* seed). Iberian *A. thaliana* populations are composed of individuals exhibiting both winter and spring annual habits, but the proportion of spring-germinated plants increases at high elevations as a result of high mortality rates of vegetative rosettes due to harsh winter conditions [49]. Flowering always occurs in late winter or early spring and fruits shed seed throughout spring and early summer [49].

**Figure 1:**
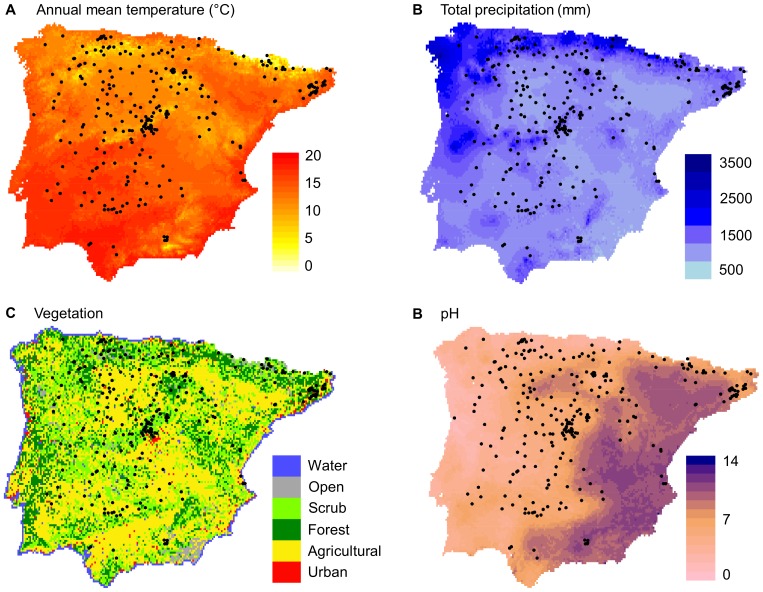
Distribution of the 279 *A. thaliana* Iberian populations of study. Maps show the geographical distribution of annual mean temperature (°C), total precipitation (mm), vegetation data, and pH.

We sampled 279 *A. thaliana* populations that were surveyed across the major part of the Iberian Peninsula (ca. 800×700 km^2^; 36.00°N –43.48°N, 3.19°E –9.30°W; Fig. 1) during the period 2004 − 2009. Populations were geo-referenced for their latitude, longitude and altitude using a GPS (Garmin International, Inc., Olathe, KS, USA), with an estimated average positional error of 4–6 m. Populations were separated by 1–1,042 km and altitudes ranged 1–2,662 m.a.s.l. For each population, we collected seeds from several individuals per population (approximately 6–20 individuals) separated by a few metres from each other. For all individuals sampled, we produced a new generation of seeds in controlled conditions in the glasshouse facilities at the Centro Nacional de Biotecnología (CNB-CSIC) in Madrid. Individuals lacking a vernalization requirement to flower completed the life cycle in 1 − 5 months in glasshouse conditions at approximately 22°C and 16 h of light. In the case of individuals requiring a cold period to induce flowering, young vegetative rosettes grown in glasshouse conditions were transferred to growth chambers at 4°C with a short-day photoperiod (8 h of light/16 h of darkness) during 6 weeks. After that time, plants were placed back in glasshouse conditions to complete the life cycle. Seeds were stored in cellophane bags at room temperature and dry conditions in darkness.

In this study, we used one individual from each of the 279 *A. thaliana* populations. We selected one individual per population, which exhibited a common phenotype within its population based on average flowering time or vernalization requirement. Furthermore, all 279 selected individuals (accessions hereafter; [53]) were genetically different from each other, as shown by genetic data obtained from a set of 250 polymorphic genome-wide neutral SNPs previously analysed in Iberian *A. thaliana* accessions [47], [48]. Genetically, the collection of 279 *A. thaliana* accessions exhibited a significant isolation-by-distance pattern (*N* = 279, *r* = 0.35, *P*<0.001; Mantel test based on geographic and genetic distance matrices), which was highly consistent with previous results on the genetic structure of Iberian *A. thaliana* populations based on 100 [46] and 182 accessions [48].

*Arabidopsis thaliana* is not an endangered or protected species across its distribution range. Field sampling was carried out in locations where no permission was required, except at Doñana National Park (permission issued by Estación Biológica de Doñana) and Sierra de Grazalema Natural Park (permission issued by Red Andaluza de Jardines Botánicos de la Consejería de Medio Ambiente y Ordenación del Territorio de la Junta de Andalucía).

### The Field Experiment

**Figure 2:**
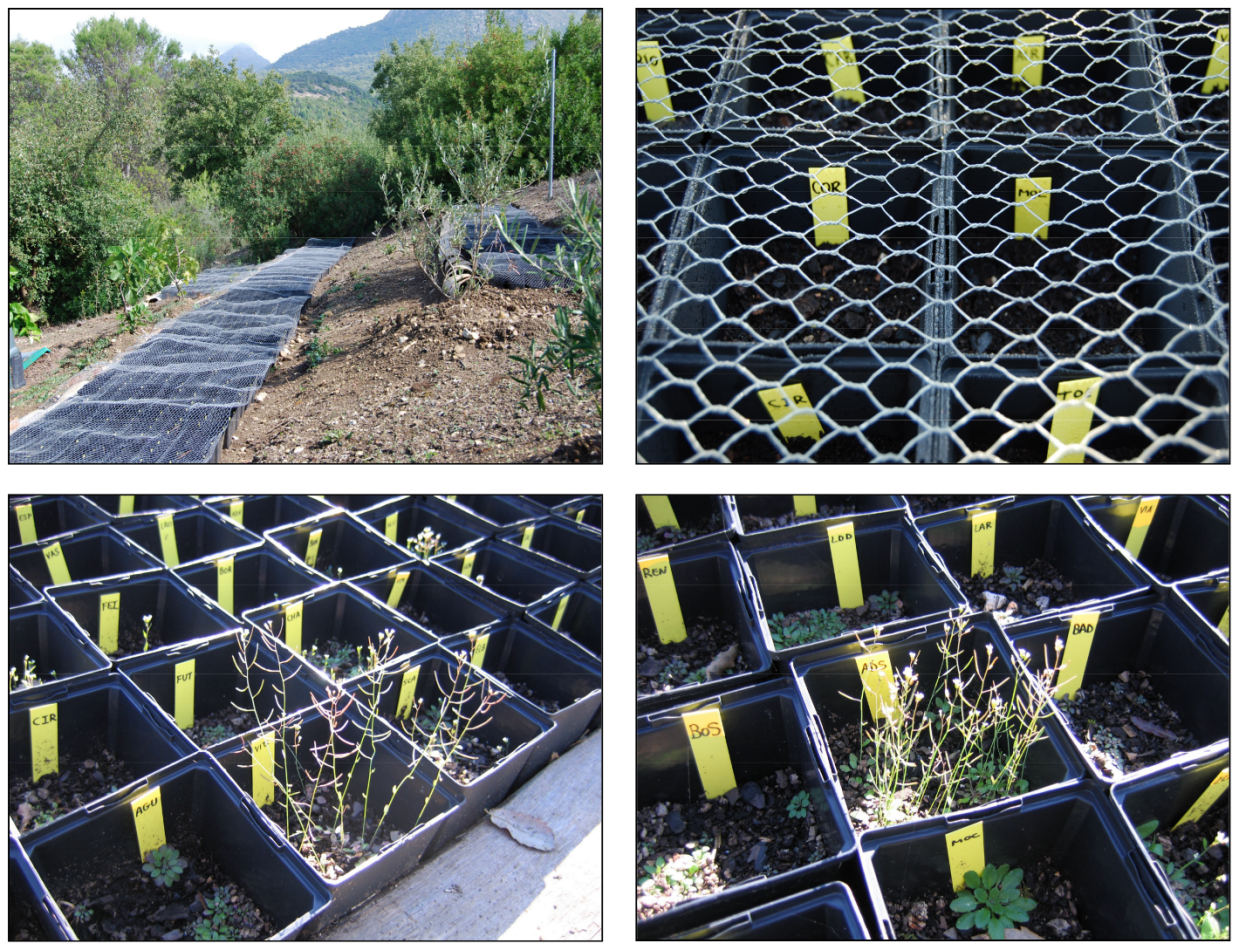
Photographs of the experimental setting at the El Castillejo Botanical Garden. Panels include a general view of the blocks and detailed views of pots with labels covered with the wire mesh, vegetative rosettes and fruiting plants of *Arabidopsis thaliana*.

In 2010, the 279 *A. thaliana* accessions were multiplied again by the single seed descent method as described above to discard possible effects of variable seed age on plant traits. For each accession, we prepared eight batches, each of them with 60 filled seeds, and stored them in 1.5 ml plastic tubes at room temperature in darkness until the sowing day. On October 6, 2010, we sowed the 60 seeds per batch in square plastic pots (12×12×12 cm^3^) filled with standard soil mixture (Abonos Naturales Cejudo Baena S.L., Utrera, Spain) at the El Castillejo Botanical Garden in Sierra de Grazalema Natural Park in SW Spain (El Bosque, Cádiz province, 36.46°N, 5.30°W, 329 m.a.s.l.). This Botanical Garden is an appropriate environment for *A. thaliana* because several accessions from environmentally contrasted Iberian locations completed the life cycle successfully there [54]. During the experiment (from October 6, 2010, to May 5, 2011), mean monthly minimum and maximum temperatures were 7.2°C and 20.4°C, respectively, and precipitation totalled 810 mm (Figure S1). Temperature data were recorded with HOBO Pendant® UA-002-08 temperature loggers (Onset Computer Corporation, Inc., Bourne, MA, USA) and precipitation was recorded daily at the meteorological station of the experimental facility. The experiment had eight blocks and each block contained one replicate per accession that was randomly placed within the block (Fig. 2). Overall, this experiment included 2,232 pots (279 accessions × 8 blocks) and 133,920 seeds (2,232 pots × 60 seeds). Blocks were covered by 2-cm wire mesh to protect young plants from bird and rodent depredation (Fig. 2).

We surveyed the experiment every 15 days to record the maximum number of vegetative rosettes per pot. In total, we conducted five surveys between October 6 and December 17, 2010. In winter, mortality of vegetative rosettes increases and recruitment of new individuals is virtually nil, rendering winter surveys as unimportant for this trait [54]. A flowering date was assigned to each pot when the majority of the individuals in the pot developed flowering buds and had the first flower open (as in [54]). It must be emphasized that all individuals within each pot were sisters obtained by self-fertilization and their reproductive behaviour was quite homogeneous. Flowering time for each pot was estimated as the number of days between seed sowing, given that seed germination peaked within the first 15 days of the experiment for all accessions, and flowering date. We counted the number of fruiting individuals per pot and the total number of fruits per individual when all individuals finished flowering and fruiting. After recording data, plants were removed and incinerated, and the soil composted to avoid uncontrolled seed dispersal at the experimental facility.

In this experiment, we monitored a maximum of 57,638 vegetative rosettes during the autumn surveys (mean ± SD = 53,054.8±3,556.3 vegetative rosettes) and recorded a total of 46,638 reproductive individuals that overall produced 554,807 fruits (grand mean ± SD = 11.9±30.6; range = 1–982 fruits per plant). The extent of flower abortion, fruit depredation, insect damage and plant disease was rather low in this experiment; only 318 (0.68%) reproductive individuals were discarded due to these factors.

### Seed Weight and Seed Germination

We estimated seed weight and seed germination for the 279 *A. thaliana* accessions. For each accession, we weighted, to the nearest 0.1 mg, three batches of 60 filled seeds each using a Sartorious BP61S balance (Sartorius AG, Göttingen, Germany). Seed weight was obtained by averaging the three measurements. In this experiment, we used a total of 50,220 seeds (279 accessions × 60 seeds × 3 batches).

We also estimated seed germination under three temperatures (10, 22 and 30°C). 10°C represents the autumn mean temperature of several Iberian *A. thaliana* populations when seed germination peaks [12], 22°C is the temperature commonly used to germinate *A. thaliana* seeds in growth chamber conditions, and 30°C is known to induce secondary seed dormancy in *A. thaliana* [55]. For each accession and temperature treatment, we conducted two germination assays that exhibited a very similar behaviour (*N* = 279; *r* = 0.83–0.86; Pearson’s correlation). For each germination assay, one batch of 50 filled seeds per accession was placed in a moist Petri dish at 4°C in darkness for four days in an AGP-700-ESP incubator (Radiber S.A., Barcelona, Spain). After that, we transferred Petri dishes to a FITOCLIMA-10.000-EH growth chamber (ARALAB, Rio de Mouro, Portugal) for five days in light and in the corresponding temperature (10, 22 or 30°C). We counted the number of germinated seeds under a Stemi-2000-C stereomicroscope (Carl Zeiss Optical, Inc., Chester, VA, USA). Seeds were scored as germinated when the root tip protruded the seed coat. For each temperature treatment, seed germination was calculated by averaging the proportion of seeds germinated between the two assays. In this experiment, we used a total of 83,700 seeds (279 accessions × 50 seeds × 3 temperatures × 2 assays).

### Environmental Data

We developed a geographic information system to generate an environmental database for the 279 geo-referenced *A. thaliana* accessions (Fig. 1) at a resolution of 1 km^2^. Weather data encompassed 19 bioclimatic variables [56], which consisted in a combination of annual trends, seasonality and extreme conditions relevant to species physiological tolerances. These variables were generated using monthly data from the Digital Climatic Atlas of the Iberian Peninsula (http://opengis.uab.es/wms/iberia/en_index.htm; [57]). Vegetation data were obtained from the CORINE Land Cover 2000 (http://www.eea.europa.eu/publications/COR0-landcover). We calculated the percentage of humanised habitat, including urban, crops and semi-natural grasslands, in a 78-ha circular area (500 m radius) around the GPS coordinates. The percentages of humanised and natural habitat, including primarily woody vegetation, were significantly negatively correlated (*N* = 279; *r* = −0.95; *P*<0.001; Pearson’s correlation) and nearly summed to 100%. Here, we only used the percentage of humanised habitat. Finally, soil data were characterised by pH, which was obtained from the Soil Geographical Database of Eurasia v.4 (http://eusoils.jrc.ec.europa.eu).

### Statistical Analyses

Life-history traits of *A. thaliana* accessions included in this study were seed weight, seed germination at three temperatures, maximum number of vegetative rosettes, flowering time, number of reproductive individuals, and mean number of fruits per individual. We also estimated a surrogate of individual fitness by multiplying the proportion of seeds becoming adult individuals times the mean number of fruits per individual. For all life-history traits, we used the mean among replicates of the different experiments. We also used replicates to estimate broad sense heritability for all life-history traits as *h^2^* = *V_G_*/(*V_G_*+*V_E_*), where *V_G_* is the among-accession variance component and *V_E_* is the residual variance.

Individuals within pots experienced asymmetric competition so that a few individuals performed much better, in terms of fruit production, than the rest of individuals in the pot. In order to avoid bias in the estimation of the reproductive output due to suppressed individuals bearing one fruit per plant, we estimated the number of fruits per individual by excluding the largest individual from each pot and averaging the number of fruits of the 15 largest individuals from all pots. Analyses conducted with different estimates for the mean number of fruits per individual (e.g. number of fruits of the largest individual, mean number of fruits of the 30 largest individuals) were highly consistent (results not shown). We considered that the 15 largest individuals captured the winners of the asymmetric competition process. In fact, the maximum number of individuals observed in the pots and the skewness of the number of fruits per individual were significantly positively correlated (*N* = 279; *r* = 0.53; *P*<0.001; Dutilleul’s *t*-test), indicating that asymmetric competition increased with density. Previous experiments with an identical set-up also showed that density only affected the number of fruits per individual whilst the rest of life-history traits remained unaffected [54].

Spatial autocorrelation patterns of environmental variables (19 weather records, percentage of humanised habitat and pH) and life-history traits (seed weight, seed germination at three temperatures, maximum number of vegetative rosettes, flowering time, number of reproductive individuals, mean number of fruits per individual, and individual fitness) were analysed with PASSaGE v.2 [58]. For each environmental variable and life-history trait, we computed Moran’s *I* autocorrelation coefficients and their significance was estimated from 1000 permutations. Correlations between pairs of environmental variables and between pairs of life-history traits were tested with Dutilleul’s modified *t*-test using SAM v.4 [59]. Dutilleul’s *t*-test corrects the variance of the test statistic and the degrees of freedom according to the extent of spatial autocorrelation of each variable [60]. We also analysed the correlation between life-history traits, excluding the two traits used to compute fitness, and individual fitness with Dutilleul’s modified *t*-test.

Simultaneous autoregressive models (SAR) were performed to test the effects of environmental variables on life-history traits using SAM. SAR is a regression technique, based on generalised least squares (GLS), to estimate regression parameters that takes spatial patterns of data into account by including an additional term for the autocorrelation matrix of the errors [61]. We did not transform any variable or trait because the lack of autocorrelation patterns in the residuals showed that the assumptions of the analyses were met. To check the robustness of patterns obtained with SAR models, we also conducted a complementary approach by performing a canonical correlation analysis (CCA) with SYSTAT v.13 (Systat Software, Inc., Chicago, IL, USA). CCA evaluates the relationship between two sets of variables and generates predicted values for each set of variables that have the highest linear correlation between them. In our case, CCA correlated the set of environmental variables and the set of life-history traits while taking among-environmental variable and among-life-history trait correlations into account. We only used weather variables showing pairwise correlation coefficients below 0.75 to avoid excessive collinearity between explicative variables [62]. As a result, we eventually included nine out of 19 weather variables in both SAR and CCA analyses. In the case of CCA, we also considered those life-history traits with pairwise correlation coefficients below 0.75 for the same reason.

Finally, for each of the nine weather variables selected, we estimated similarity matrices based on Euclidian distances. We also estimated similarity distances for life-history traits among accessions. Parametric partial Mantel tests were conducted with PASSaGE to analyse the relationship between climatic similarity and life-history trait similarity controlling for the geographic position of accessions given by the geographic distance matrix. In order to understand the relationship between phenotypic, genetic and environmental variation in this set of Iberian *A. thaliana* accessions, we also conducted partial Mantel tests to assess the relationship between genetic similarity and life-history trait similarity, and between genetic similarity and climatic similarity, always controlling for the geographic position of accessions.

## Results

### Patterns of Variation in Environmental Variables

The main environmental features of the 279 *A. thaliana* populations of study were as follows. Annual mean temperature ranged between 4.9 and 18.2°C (mean ± SD = 12.2±2.6°C; Fig. 1A), the minimum mean temperature of the coldest month varied from a low of −7.5°C to a high of 7.3°C (mean ± SD = 0.05±2.6°C), and the maximum mean temperature of the warmest month varied between 20.6°C and 35.9°C (mean ± SD = 29.1±3.4°C). Total annual precipitation ranged between 384.3 and 1,799.4 mm (mean ± SD = 771.3±281.6 mm; Fig. 1B), precipitation of the driest month varied between 0.6 and 90.3 mm (mean ± SD = 24.8±17.5 mm), and precipitation of the wettest month ranged from a low of 44.1 to a high of 288.4 mm (mean ± SD = 103.1±39.3 mm). The percentage of humanised habitat ranged between 0 and 100% (mean ± SD = 40.3±37.2%; Fig. 1C) and pH varied between the acidic 3.6 and the basic 7.5 (mean ± SD = 5.7±0.8; Fig. 1D). In general, correlations between pairs of environmental variables indicated that accessions from environments with warmer annual mean temperature were those with lower precipitations during the dry season, more seasonal precipitations throughout the year, and a higher percentage of humanised habitat (Table S1). Accessions from environments with basic soils were also those with lower annual precipitations (Table S1).

All 19 climatic variables were significantly spatially autocorrelated (range of Moran’s *I* = 0.19–0.36; *P*<0.001 in all cases) in this set of *A. thaliana* accessions. The pH also exhibited a significant spatial autocorrelation (Moran’s *I* = 0.20; *P*<0.001). The percentage of humanised habitat was not significantly spatially autocorrelated (*P* = 0.18). The distances between population pairs exhibiting significant autocorrelation patterns for environmental variables varied approximately between 130 and 350 km.

### Patterns of Variation in Life-history Traits

**Figure 3:**
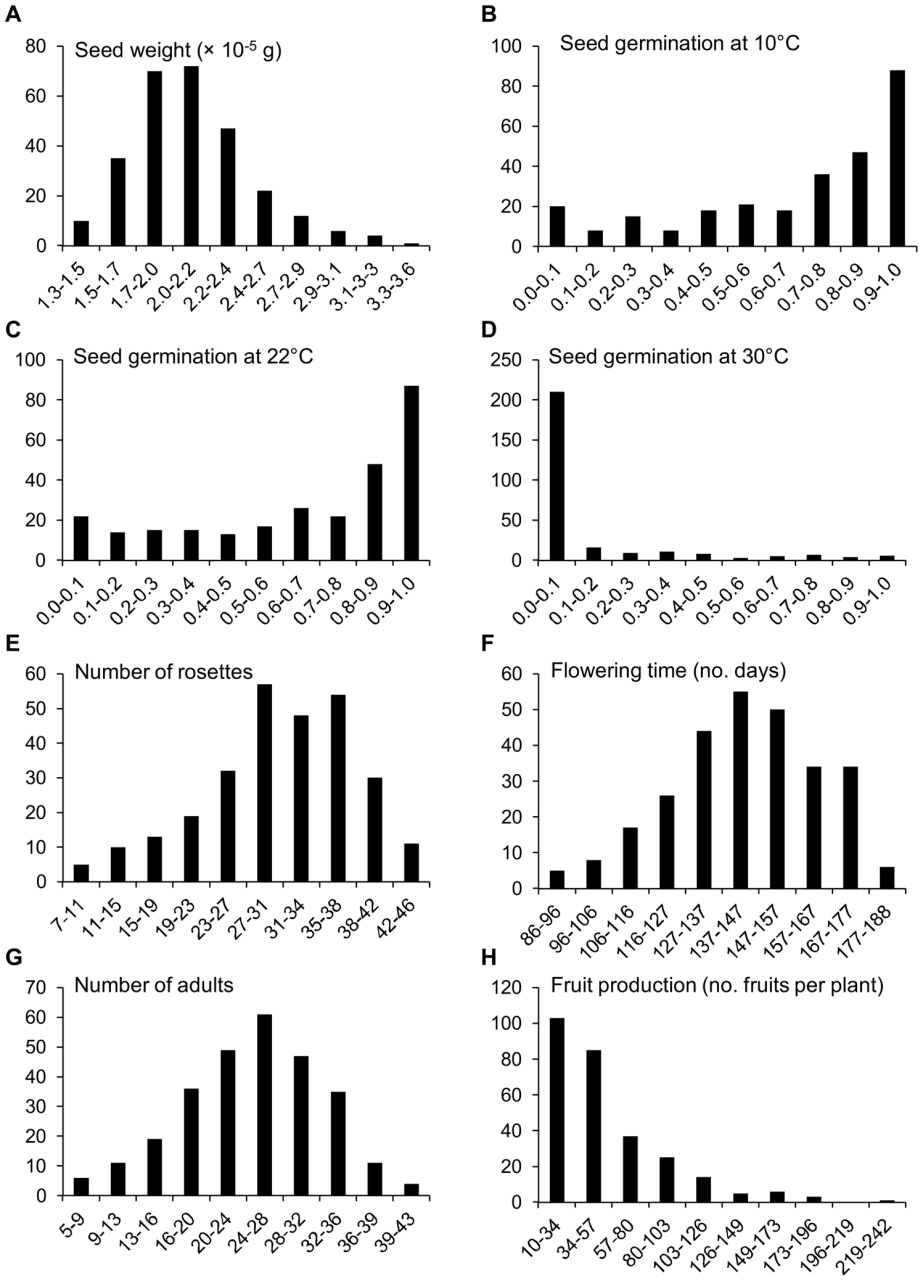
Frequency distributions for life-history traits of *A. thaliana*. Graphs depict the number of accessions within each interval. *N* = 279 accessions in all cases.

In our set of 279 *A. thaliana* accessions, seed weight (mean ± SD = 2.1±0.4×10^−5^; range = 1.3–3.6×10^−5^ g; Fig. 3A) was not significantly correlated with any life-history trait (Table 1). All seed germination proportions at three temperatures were significantly positively correlated among them (Table 1). Seed germination at 10°C (mean ± SD = 0.69±0.29; Fig. 3B) and 22°C (mean ± SD = 0.67±0.31; Fig. 3C) showed high proportions, whereas seed germination at 30°C (mean ± SD = 0.12±0.23; Fig. 3D) exhibited the lowest value. Seed germination at 10°C and 22°C were significantly positively correlated with number of vegetative rosettes (mean ± SD = 30.1±8.1; range = 8.9–46.3 rosettes per pot; Fig. 3E) and number of reproductive individuals (mean ± SD = 24.8±7.4; range = 4.8–43.3 individuals per pot; Table 1 and Fig. 3F). The number of vegetative rosettes and the number of reproductive individuals were strongly positively correlated (Table 1). Finally, flowering time (mean ± SD = 143.1±20.4; range = 85.6–187.5 days; Fig. 3G) was significantly positively correlated with mean number of fruits per individual (mean ± SD = 53.6±37.3; range = 10.3–242.1 fruits per individual; Table 1 and Fig. 3H).

**Table 1:** Pearson’s correlation coefficients among life-history traits and heritability values of 279 Iberian *A. thaliana* accessions.

	Seedweight	Germination(10°C)	Germination(22°C)	Germination(30°C)	Number ofrosettes	Floweringtime	Number ofadults	Fruitproduction
Seed weight	0.96							
Germination (10°C)	−0.116 *ns*	0.87						
Germination (22°C)	−0.074 *ns*	0.672***	0.82					
Germination (30°C)	−0.053 *ns*	0.319***	0.416***	0.86				
Number of rosettes	0.096 *ns*	0.344***	0.240***	0.185 *ns*	0.41			
Flowering time	0.106 *ns*	−0.177 *ns*	−0.004 *ns*	0.033 *ns*	−0.006 *ns*	0.93		
Number of adults	0.072 *ns*	0.341***	0.211***	0.144 *ns*	0.922***	−0.107 *ns*	0.24	
Fruit production	−0.059 *ns*	0.056 *ns*	0.157 *ns*	0.139 *ns*	0.112 *ns*	0.418***	0.048 *ns*	0.08

Heritability values are given on the diagonal. Correlation coefficients were obtained from Dutilleul’s modified *t*-tests. A new threshold significance value (α = 0.0018) was set after applying the Dunn-Šidák correction (1– [1– α] ^1/n^) for multiple comparisons. Significance: ***P<0.0001, *ns*; non-significant.

Individual fitness was significantly positively correlated with seed germination at 10°C (*r* = 0.17; *P* = 0.0004), seed germination at 22°C (*r* = 0.22; *P*<0.0001), seed germination at 30°C (*r* = 0.19; *P* = 0.001), number of vegetative rosettes (*r* = 0.41; *P*<0.0001), and flowering time (*r* = 0.33; *P*<0.0001). In contrast, individual fitness and seed weight were not significantly correlated (*P* = 0.67).

Our estimates of heritability for all life-history traits indicated high values for seed weight, flowering time, and the three seed germination values at three temperatures (range *h^2^* = 0.82–0.96; Table 1). The number of vegetative rosettes, the number of reproductive individuals and the mean number of fruits per individual exhibited intermediate or low heritability values (range *h^2^* = 0.08–0.41; Table 1). Finally, heritability for individual fitness was also low in this set of *A. thaliana* accessions (*h^2^* = 0.09).

Only seed weight (Moran’s *I* = 0.33; *P*<0.001) and flowering time (Moran’s *I* = 0.21; *P*<0.001) showed significant spatial autocorrelation patterns whereas the rest of traits were not significantly spatially autocorrelated (*P*>0.63 in all cases). Individual fitness was not spatially autocorrelated either (*P* = 0.91). The distance between accession pairs with significant autocorrelation patterns was approximately of 500 km for seed weight and 300 km for flowering time.

### Relationship between Environmental Variables and Life-history Traits

Univariate SAR models showed that only seed weight and flowering time were significantly correlated with environmental variables (Table 2). Seed weight was significantly negatively correlated with annual mean temperature, mean temperature of the wettest quarter and the percentage of humanised habitat, whereas it was significantly positively correlated with annual precipitation (Table 2). Flowering time was significantly negatively correlated with annual mean temperature, mean temperature of the wettest quarter, mean temperature of the driest quarter, precipitation seasonality and the percentage of humanised habitat (Table 2). In contrast, flowering time was significantly positively correlated with precipitation of the driest month and pH. It is noteworthy that annual mean temperature exhibited the highest correlation coefficients with seed weight and flowering time (Table 2).

**Table 2:** Correlation coefficients between life-history traits and environmental variables in *A. thaliana*.

	Seedweight	Germination(10°C)	Germination(22°C)	Germination(30°C)	Number ofrosettes	Floweringtime	Number ofadults	Fruitproduction
Annual temperature	−0.391***	0.064 *ns*	−0.135 *ns*	−0.051 *ns*	−0.194 *ns*	−0.699***	−0.086 *ns*	−0.233 *ns*
Diurnal range	−0.137 *ns*	−0.006 *ns*	0.095 *ns*	0.053 *ns*	−0.086 *ns*	0.205 *ns*	−0.089 *ns*	0.133 *ns*
Temp. seasonality	−0.002 *ns*	−0.068 *ns*	0.097 *ns*	−0.013 *ns*	−0.067 *ns*	0.073 *ns*	−0.079 *ns*	0.100 *ns*
Temp. wettest quarter	−0.287***	−0.032 *ns*	−0.141 *ns*	0.049 *ns*	−0.125 *ns*	−0.385***	−0.071 *ns*	−0.073 *ns*
Temp. driest quarter	−0.220 *ns*	0.099 *ns*	−0.076 *ns*	−0.070 *ns*	−0.160 *ns*	−0.427***	−0.133 *ns*	−0.118 *ns*
Annual precipitation	0.225***	0.045 *ns*	0.018 *ns*	−0.018 *ns*	0.155 *ns*	0.070 *ns*	0.132 *ns*	−0.012 *ns*
Prec. driest month	0.278 *ns*	−0.023 *ns*	−0.014 *ns*	0.086 *ns*	0.131 *ns*	0.307***	0.041 *ns*	0.117 *ns*
Prec. seasonality	−0.092 *ns*	0.091 *ns*	−0.030 *ns*	−0.097 *ns*	0.035 *ns*	−0.506***	0.056 *ns*	−0.258 *ns*
Humanised habitat	−0.241***	0.141 *ns*	0.061 *ns*	0.087 *ns*	0.041 *ns*	−0.262***	0.084 *ns*	0.018 *ns*
pH	0.098 *ns*	−0.149 *ns*	−0.056 *ns*	−0.097 *ns*	−0.038 *ns*	0.225***	−0.055 *ns*	0.049 *ns*

Correlation coefficients were obtained from SAR models. A new threshold significance value (α = 0.0006) was set after applying the Dunn-Šidák correction (1– [1– α] ^1/n^) for multiple comparisons. Significance: ***; *P*<0.0001, *ns*; non-significant.

**Figure 4:**
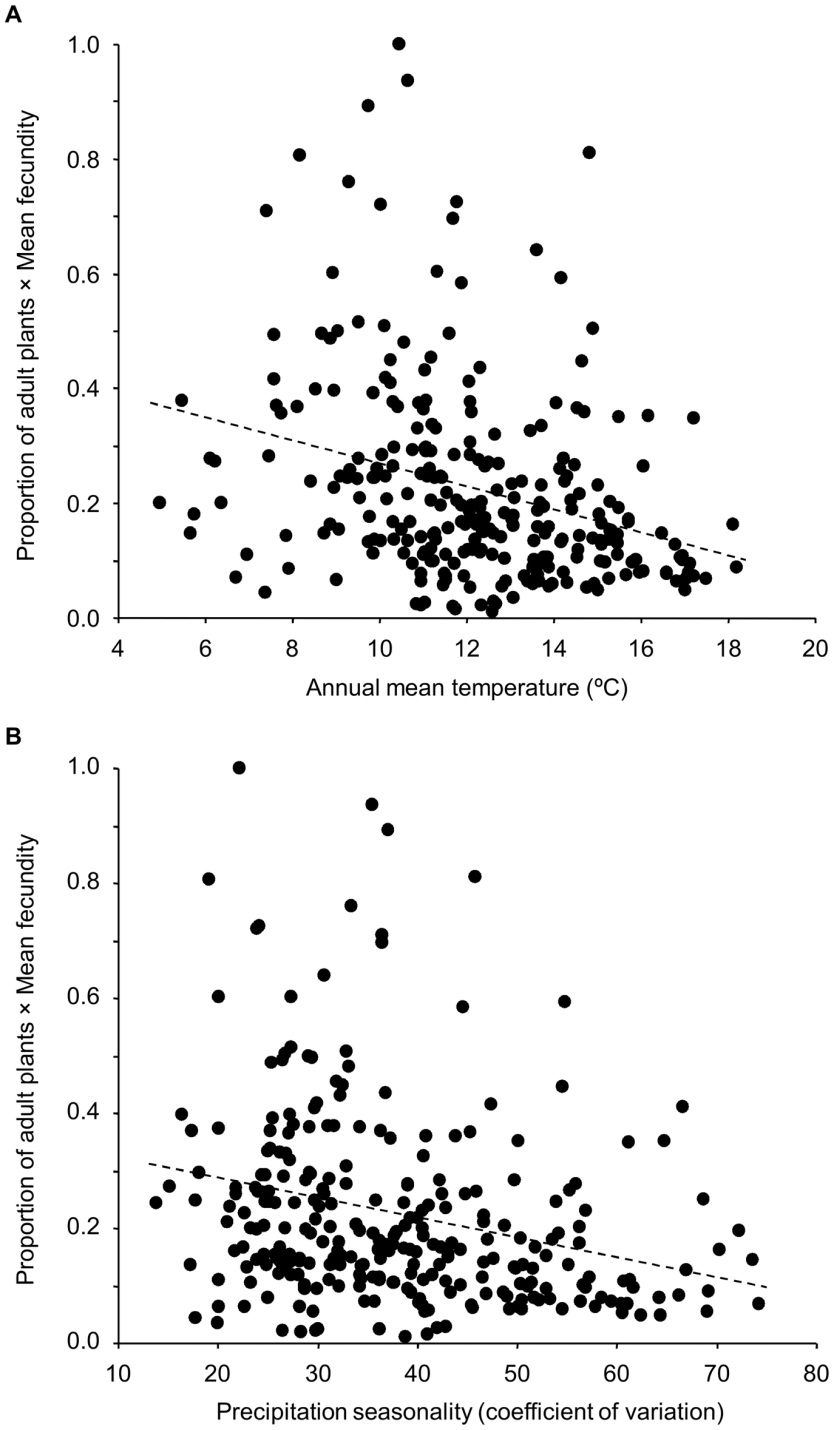
Relationship between individual fitness and annual mean temperature or precipitation seasonality in *A. thaliana*.

SAR models also indicated that individual fitness was significantly negatively correlated with annual mean temperature (*r* = −0.23; *P* = 0.003) and precipitation seasonality (*r* = −0.23; *P* = 0.011). This indicates that *A. thaliana* accessions from warmer environments and environments with higher seasonality in precipitation regimes, which were also positively correlated between them (Table S1), performed worse at the experimental facility (Fig. 4). Individual fitness was not significantly correlated with any other climatic variable (*P*>0.07 in all cases). Despite the significant negative linear trend between individual fitness and annual mean temperature, accessions from populations with annual mean temperatures below 7.5°C (*N* = 45 accessions; 16.1%) and above 15°C (*N* = 20 accessions; 7.2%) clearly showed reduced fitness (Fig. 4A).

CCA yielded two significant overall canonical correlation coefficients (Table 3), although the first canonical correlation coefficient was about two-fold higher than the second one (coefficients = 0.83 and 0.36, *P*<0.008 in both cases; Table 3). This indicated the preponderance of the first canonical correlation coefficient in this analysis. The most important canonical variate (i.e. a linear combination of environmental variables and life-history traits in this case) showed that only flowering time and annual mean temperature exhibited a negative relationship (Table 3). The second canonical variate showed the positive relationship between seed weight and number of vegetative rosettes, and the negative relationship of these two traits with mean number of fruits per individual (Table 3). Such life-history traits were in turn negatively related to mean diurnal range and the percentage of humanised habitat, whereas they were positively related to annual precipitation (Table 3). Overall, CCA results highlighted the strong negative relationship between flowering time and annual mean temperature in this set of Iberian *A. thaliana* accessions.

**Table 3:** Canonical correlations between environmental variables and life-history traits in *A. thaliana*.

		1st Canonical	2nd Canonical
Group	Variables	variate correlation	variate correlation
Life history	Seed weight	0.35	**0.83**
	Germination (10°C)	−0.10	−0.08
	Number of rosettes	0.19	**0.38**
	Flowering time	**0.95**	−0.28
	Fruit production	0.36	−**0.40**
Environment	Annual temperature	−**0.93**	−0.10
	Diurnal range	0.10	−**0.56**
	Temp. seasonality	0.17	−0.24
	Temp. wettest quarter	−0.46	−0.12
	Temp. driest quarter	−0.60	−0.21
	Annual precipitation	0.17	**0.59**
	Prec. driest month	0.42	0.24
	Prec. seasonality	−0.54	0.26
	Humanised habitat	−0.46	−**0.36**
	pH	0.23	0.12

Results for the first two significant canonical variates are given. Overall canonical correlations for the first and second canonical variates are 0.83 and 0.36, respectively. Canonical correlations of environmental variables and life-history traits above the overall canonical correlation for each canonical variate are in bold face.

Partial Mantel tests correlating climatic similarity with similarity in life-history traits also showed that only seed weight and flowering time exhibited significant correlations (Table 4). Similarity in seed weight and flowering time was significantly positively correlated with similarity between environments for annual mean temperature (Table 4). Similarity in seed weight and flowering time was also significantly positively correlated with similarity between environments for mean temperature of the wettest quarter and precipitation seasonality, respectively (Table 4). These analyses provided complementary support to the relationship inferred from SAR and CCA models between flowering time and annual mean temperature, and to a lesser extent between seed weight and annual mean temperature. Finally, none of the partial Mantel tests correlating similarity between environments and similarity in individual fitness was significant (*P*>0.12 in all cases).

**Table 4:** Correlation coefficients between life-history trait and environmental similarity in *A. thaliana*.

	SeedWeight	Germination(10°C)	Germination(22°C)	Germination(30°C)	Number ofrosettes	Floweringtime	Number ofadults	Fruitproduction
Annual temperature	0.185**	0.009 *ns*	−0.011 *ns*	−0.007 *ns*	0.010 *ns*	0.429**	−0.040 *ns*	0.044 *ns*
Diurnal range	0.055 *ns*	0.018 *ns*	0.029 *ns*	−0.068 *ns*	0.024 *ns*	−0.011 *ns*	0.047 *ns*	−0.033 *ns*
Temp. seasonality	−0.031 *ns*	0.030 *ns*	0.077 *ns*	−0.048 *ns*	0.039 *ns*	0.051 *ns*	0.045 *ns*	−0.011 *ns*
Temp. wettest quarter	0.155**	0.007 *ns*	−0.023 *ns*	−0.047 *ns*	−0.048 *ns*	0.070 *ns*	−0.054 *ns*	0.018 *ns*
Temp. driest quarter	−0.015 *ns*	0.018 *ns*	−0.048 *ns*	0.089 *ns*	0.113 *ns*	0.096 *ns*	0.066 *ns*	0.032 *ns*
Annual precipitation	0.092 *ns*	−0.037 *ns*	−0.019 *ns*	−0.034 *ns*	0.027 *ns*	−0.036 *ns*	0.021 *ns*	−0.033 *ns*
Prec. driest month	0.019 *ns*	0.005 *ns*	0.035 *ns*	0.024 *ns*	0.087 *ns*	0.045 *ns*	0.066 *ns*	−0.027 *ns*
Prec. seasonality	−0.007 *ns*	−0.011 *ns*	0.031 *ns*	−0.004 *ns*	−0.021 *ns*	0.181**	−0.034 *ns*	0.033 *ns*

Correlation coefficients were obtained from partial Mantel tests. A new threshold significance value (α = 0.001) was set after applying the Dunn-Šidák correction (1– [1– α] ^1/n^) for multiple comparisons. Significance: **; *P*<0.001, *ns*; non-significant.

Additional partial Mantel tests between genetic similarity and environmental similarity yielded significant positive correlations for almost all environmental variables analysed in this study, although weaker than Mantel tests for life-history traits (range *r* = 0.04–0.10, *P*<0.035 in all cases). Only similarity in annual mean temperature, precipitation seasonality, and pH did not show significant correlations with genetic similarity (*P*>0.08 in all cases). The strong genetic structure of *A. thaliana* in the Iberian Peninsula, as reported elsewhere [46], [48], accounts for the high similarity between genetic and environmental distances found in this study. Finally, genetic similarity was significantly positively correlated with similarity in seed weight, flowering time, mean number of fruits per individual and individual fitness (range *r* = 0.06–0.11, *P*<0.001 in all cases). However, these correlations were again weaker than those between life-history traits and environmental variables.

## Discussion

Understanding large-scale adaptive variation requires the measurement of changes in phenotypic traits, the effects of such changes on fitness, and the identification of the environmental factors acting as proxies of selective agents. Overall, phenotypic variation is expected to move towards different phenotypes that best fit different environments, as developed by [63] and recently reviewed by [64], configuring the Wright’s adaptive landscapes if phenotypes represent different gene combinations [65], [66]. In this study, we have used the annual plant *Arabidopsis thaliana* to unravel the adjustment between environment and life history, highlighting the importance that the geographical component has to identify ecologically and evolutionarily relevant phenotypic traits and environmental factors. Geographically-explicit approaches evaluating the match between organisms and the environments where they live can be very important to assess and predict the consequences of dramatic environmental changes, such as global warming and increasing aridity.

### Variation in Environmental Variables and Life-history Traits

The analyses performed in this study indicated that the main adjustment between *A. thaliana*’s life history and the environmental conditions found across the Iberian Peninsula was mainly carried out through variation in flowering time. In addition, geographical variation in flowering time appeared to be related to geographical variation in annual mean temperature. These patterns of variation in flowering time are in agreement with recent empirical evidence indicating that flowering in annuals [29], [30], [32] and perennials [29], [67]–[72] takes place earlier as environments get warmer. Such generalised advance of reproductive phenology with increasing temperature occurs because reductions in the life cycle duration allow plants to escape from unfavourable environmental conditions for growth and reproduction [73], [74]. It must be emphasized, however, that other traits correlated with flowering time, but not measured in this study, could also represent targets of selection, e.g. rosette size at bolting or leaf production rate during vegetative growth [75]. Further field experiments are needed to take into account architectural plant traits to be compared with life-history traits.

Our results showed that *A. thaliana* accessions with more vigorous seed germination, higher recruitment and later flowering times exhibited higher individual fitness at the experimental facility. We have recently shown that the environmental conditions at the El Castillejo Botanical Garden correspond quite well to environments with mild and moderately cold winters [54]. Such environments activate the vernalization pathway, i.e. the flowering inductive effect of low temperature during winter months on vegetative rosettes, which is in turn associated to late-flowering behaviours in *A. thaliana* [48], [54]. Hence, the better fitness performance of late-flowering *A. thaliana* accessions at the experimental facility could reflect their adaptation to the environments where they come from and the good match between local and experimental climatic characteristics for these accessions. The view that the patterns of variation in flowering time found in this study are adaptive is supported by the results of the partial Mantel test, indicating that a higher climatic similarity between local environments was significantly positively correlated with higher similarity in flowering time. Recent findings also indicated that Iberian *A. thaliana* populations were more genetically differentiated for flowering time than for neutral molecular markers, suggesting that flowering time is likely to be under divergent selection in *A. thaliana* [54]. It must be noted, however, that environmental adaptations of *A. thaliana* mediated by changes in flowering time must also be influenced, and probably enhanced, by the demographic history of accessions in the Iberian Peninsula, as indicated by the significant correlations between combinations of environmental, genetic, and flowering time similarity matrices.

Seed weight was the other trait showing a significant spatial autocorrelation pattern as well as significant relationships with environmental variables. In particular, accessions from warmer environments produced lighter seeds. As shown by previous observations in *A. thaliana* [14], [76], variation in seed weight might partly be determined by variation in flowering time, since early flowering reduces the length of vegetative growth and subsequently the amount of resources that mother plants allocate to seeds. On top of that, it has also been shown that inflorescences are more effective than rosettes fixing carbon and increasing instantaneous water use efficiency in *A. thaliana* [77]. As a result, late-flowering individuals can possess the physiological means to increase carbon acquisition during the reproductive phase and eventually produce heavier seeds. Nonetheless, the lack of relationship between flowering time and seed weight found in this study suggests the existence of other factors, such as the known trade-off between seed size and seed number in *A. thaliana* [14], [78], which need to be taken into account in future experiments to better assess the relationship between life-history traits and seed production in field environments.

Finally, individual fitness at the experimental facility decreased in *A. thaliana* accessions from environments with warmer annual mean temperatures and higher seasonal rains. Interestingly, genetic similarity and individual fitness similarity were also significantly positively correlated, suggesting that the genetic background of accessions also influenced their performance in the field experiment. Despite such relationships, it is noteworthy that accessions from environments with more extreme annual mean temperatures (i.e. 15°C< annual mean temperature <7.5°C) exhibited a trend toward reduced fitness. We have already seen the expected effects of warm environments on flowering time, but an explanation is due for the lower fitness performance found for accessions from cold environments, which are generally located at high altitudes in the Iberian Peninsula [46], [48]. It has recently been shown that high-altitude *A. thaliana* populations are predominantly composed of spring-germinated plants that overwinter as seeds in the soil seed bank. This has been interpreted as an escape strategy to mitigate the effects of massive mortality of rosettes during the cold harsh winter on population viability [49]. Thus, the lower fitness performance of accessions from cold environments at the experimental facility might reflect the extent of local adaptation of such accessions to their spring annual behaviour.

### Expected Effects of Global Warming on A. Thaliana’s Life History

We want to discuss the results of this study in a context of global warming given the current great concern on this issue [79]–[81]. Based on our findings and the evidence about the effects of increased temperature on flowering time, we expect *A. thaliana* to shift its life history toward early-flowering behaviour with smaller seed sizes with increasing warming. However, it must be emphasized that *A. thaliana* is very rare in vast areas across the driest and hottest regions of the Iberian Peninsula, including lowlands from southern and eastern Spain (Fig. 1). Hence, we hypothesize a more pessimistic scenario in which *A. thaliana* might go extinct in those regions that resulted more affected by increasing warming and drought. In dry hot environments, advancing flowering time might not be enough to maintain viable populations and *A. thaliana* should acquire other traits, such as delayed seed germination, as shown in desert annuals along gradients of aridity and climate predictability [27]. Additional experiments are also needed to assess realistically the adaptive potential and the developmental constraints of the species in dry environments.

Recently, we have shown the effect of loss- and change-of-function alleles of some well-known flowering genes, such as *FRIGIDA*, *FLOWERING LOCUS C*, and *PHYTOCHROME C* on flowering time variation of Iberian *A. thaliana* accessions [48], [82]. It is becoming increasingly evident that nucleotide polymorphisms of genes involved in *A. thaliana* natural variation for flowering generally have a low frequency and are strongly geographically structured [39], [48], [53], [82], [83]. These results suggest that the role that regulatory pathways play to account for flowering time variation also vary geographically across the Iberian Peninsula. For example, accessions carrying specific alleles of *FLOWERING LOCUS C* (i.e. vernalization pathway for flowering) or *CRYPTOCHROME2* (i.e. photoperiod pathway for flowering) mainly occur in northern wet or south-western dry areas of the Iberian Peninsula, respectively [48]. Therefore, the adaptive potential of flowering time in *A. thaliana* across different Iberian regions might strongly be constrained by the differences in the genetic basis of the regulatory pathways accounting for flowering in each region. The effect of increased warming and drought on flowering time variation regulated by different genetic pathways seems difficult to predict and represents an important future research line.

### Conclusions

We stress the need to conduct experiments with extensive collections of geo-referenced accessions to evaluate the interplay between environmental factors and phenotypic variation. The reasons are manifold. First, environmental main selective pressures, although with some exceptions (e.g. pathogens or competition), vary geographically in a continuous fashion. Hence, experiments ought to capture such continuous adaptive landscape in order to be realistic and conclusive. Second, the increasing evidence that the genetic regulatory pathways underlying phenotypic variation are also geographically structured should also warn us against the use of a limited number of populations in experimental designs. Third, given the inherent spatial patterns of variation in environmental variables and life-history traits, statistical analyses need to take spatial autocorrelation into account, and only extensive spatially-explicit data sets can cope with spatial autocorrelation properly [84]. Finally, although the ideal experimental design should include the replication of the experiment in different environments [37], [85], the combination of spatially-explicit statistical tools proved to be a useful method to depict patterns of adaptive variation. Overall, merging environmental, phenotypic and molecular data using extensive geo-referenced collections of natural populations can strongly accelerate the pace with which we obtain comprehensive insights into adaptive variation in plants.

## Supporting Information

Figure S1 Daily weather records at the experimental facility during the experiment.
(TIF)

Table S1 (DOCX)
