# Supplementary figures and images for: Deciphering the Adjustment between Environment and Life History in Annuals: Lessons from a Geographically-Explicit Approach in Arabidopsis thaliana
Source: PLoS One. 2014 Feb 3;9(2):e87836. doi: 10.1371/journal.pone.0087836 (PMC3912251; doi:10.1371/journal.pone.0087836)

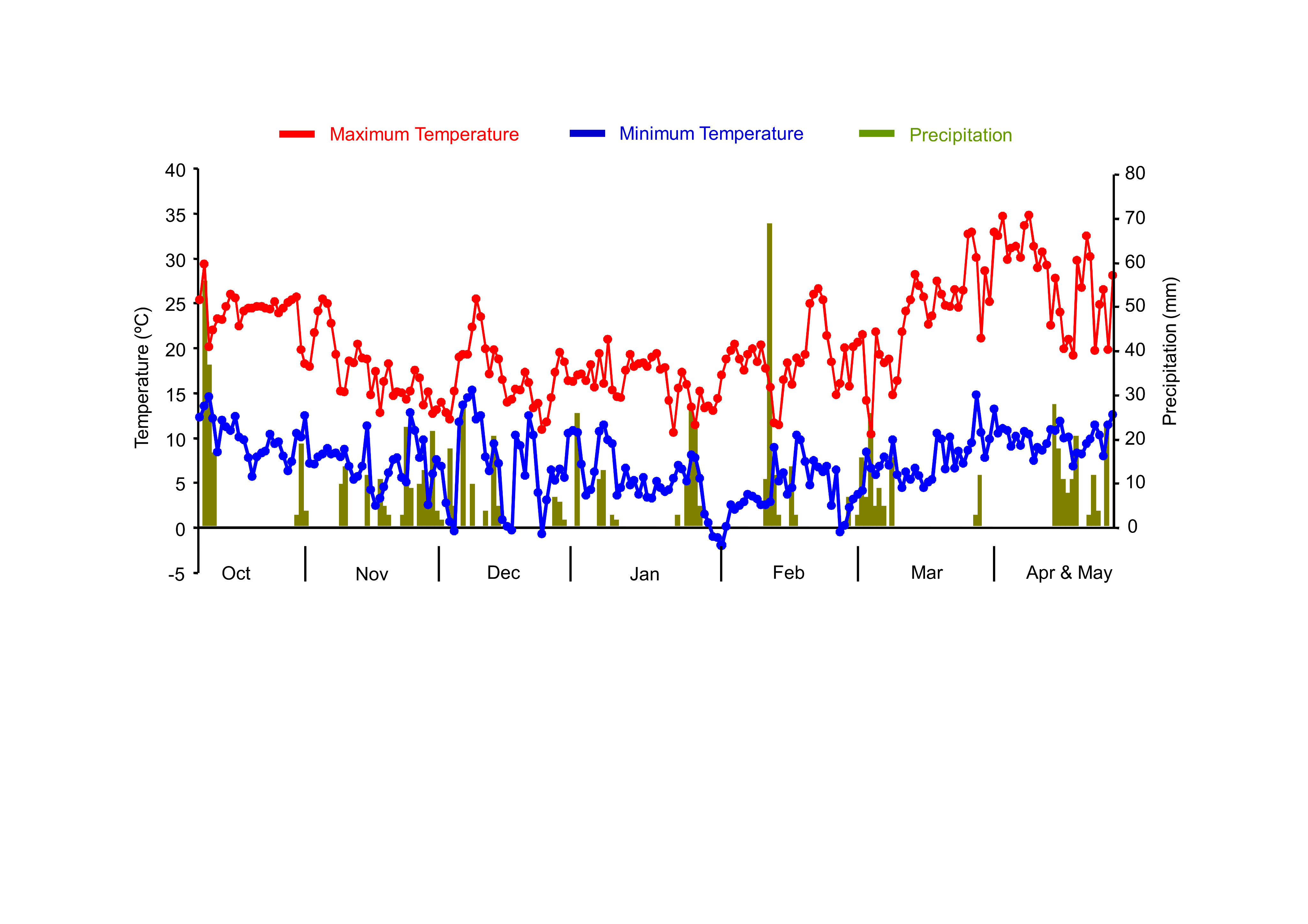

Supplement: Figure S1 — Daily weather records at the experimental facility during the experiment. (TIF) [file pone.0087836.s001.tif]
