# Supplementary material for: Deciphering the Adjustment between Environment and Life History in Annuals: Lessons from a Geographically-Explicit Approach in Arabidopsis thaliana
Source: PLoS One. 2014 Feb 3;9(2):e87836. doi: 10.1371/journal.pone.0087836 (PMC3912251; doi:10.1371/journal.pone.0087836)
Supplement: Table S1 — (DOCX) [file pone.0087836.s002.docx]

**Supplementary Table S1.** Pearson's correlation coefficients among environmental variables for the collection of 279 Iberian *Arabidopsis thaliana* populations.

|  | BIO1 | BIO2 | BIO4 | BIO8 | BIO9 | BIO12 | BIO14 | BIO15 | HUMAN | pH |
| --- | --- | --- | --- | --- | --- | --- | --- | --- | --- | --- |
| BIO1 | – |  |  |  |  |  |  |  |  |  |
| BIO2 | 0.09 *ns* | – |  |  |  |  |  |  |  |  |
| BIO4 | 0.12 *ns* | 0.64 *** | – |  |  |  |  |  |  |  |
| BIO8 | 0.48 *ns* | -0.16 *ns* | -0.07 *ns* | – |  |  |  |  |  |  |
| BIO9 | 0.72 *** | 0.06 *ns* | 0.17 *ns* | 0.05 *ns* | – |  |  |  |  |  |
| BIO12 | -0.35 *ns* | -0.50 *** | -0.56 ** | -0.28 *ns* | -0.32 *ns* | – |  |  |  |  |
| BIO14 | -0.58 ** | -0.50 ** | -0.57 *ns* | 0.04 *ns* | -0.64 **** | 0.67 *** | – |  |  |  |
| BIO15 | 0.56 ** | 0.28 *ns* | 0.26 *ns* | -0.20 *ns* | 0.55 *ns* | -0.08 *ns* | -0.73 ** | – |  |  |
| HUMAN | 0.30 ** | 0.07 *ns* | -0.08 *ns* | 0.06 *ns* | 0.23 *ns* | -0.19 *ns* | -0.19 *ns* | 0.13 *ns* | – |  |
| pH | -0.06 *ns* | 0.16 *ns* | 0.37 *ns* | 0.29 *ns* | -0.19 *ns* | -0.40 *** | -0.07 *ns* | -0.20 *ns* | -0.13 *ns* | – |

Significance: ***; *P* < 0.0001, *ns*; non-significant. Variables: BIO1 (Annual Mean Temperature), BIO2 (Mean Diurnal Range), BIO4 (Temperature Seasonality), BIO8 (Mean Temperature of Wettest Quarter), BIO9 (Mean Temperature of Driest Quarter), BIO12 (Annual Precipitation), BIO14 (Precipitation of Driest Month), BIO15 (Precipitation Seasonality), HUMAN (percentage of humanised habitat). Correlation coefficients were obtained from Dutilleul’s modified *t*-tests. A new threshold significance value (α = 0.0011) was set after applying the Dunn-Šidák correction (1 – [1 – α] 1/n) for multiple comparisons.
